# Effects of 6 weeks of complex training on athletic performance and post-activation performance enhancement effect magnitude in soccer players: a cross-sectional randomized study

**DOI:** 10.5114/biolsport.2025.139849

**Published:** 2024-08-08

**Authors:** Michał Krzysztofik, Jakub Jarosz, Robert Urbański, Piotr Aschenbrenner, Petr Stastny

**Affiliations:** 1 Institute of Sport Sciences, The Jerzy Kukuczka Academy of Physical Education in Katowice, Poland; 2 Department of Sport Games, Faculty of Physical Education and Sport, Charles University in Prague, Prague, Czech Republic; 3 Department of Biomechanics and Sports Engineering, Gdansk University of Physical Education and Sport, Gdańsk, Poland

**Keywords:** Resistance training, Power output, Countermovement jump, Broad jump, Back squat, Hip thrust

## Abstract

This study aimed to determine the effect of complex training (CT) on post-activation performance enhancement (PAPE) effect magnitude, 5- and 30-m linear sprint, 5-0-5 change-of-direction (COD), back squat (BS) and hip thrust (HT) one-repetition maximum [1RM], and jumping performance (countermovement jump [CMJ], drop jump [DJ], and broad jump [BJ]). The PAPE effect was elicited before and after each intervention by 3 BS repetitions at 90% 1RM and verified by CMJ performance. Twenty-four soccer players were randomly and equally assigned to 6 weeks of either medium (MED; [65–70%1RM]) or high-intensity (HIGH; [80–85%1RM]) CT performed twice a week. The HIGH group significantly improved their 5-m time (p < 0.001; effect size [ES] = 1.91), 30-m time (p = 0.001; ES = 0.66), BS 1RM (p = 0.019; ES = 0.19) and HT 1RM (p = 0.035; ES = 0.26), BJ length (p = 0.012; ES = 0.62) and DJ height (p = 0.002; ES = 0.57) from pre- to post-intervention. The MED group significantly improved their 5-m time (p = 0.004; ES = 0.52), BS 1RM (p = 0.019; ES = 0.36) and BJ length (p = 0.012; ES = 0.7). Significantly shorter 5-m sprint time (p = 0.001; ES = 1.63) and greater DJ height percentage increase (p < 0.001; ES = 1.81) were found in the HIGH group compared to the MED group. Moreover, a significant main effect of the group, indicating a higher PAPE response in the MED group compared to the HIGH group for CMJ peak power output, was observed at both pre- and post-CT intervention (p = 0.045; *η*^2^ = 0.171). Six weeks of either medium or high-intensity CT could be used to enhance jumping performance, linear speed and lower-body maximum strength among soccer players. Superior improvements in acceleration and DJ might be expected after high-intensity CT than medium intensity. Medium-intensity CT can improve PAPE response.

## INTRODUCTION

Complex training (CT) describes a training method where movement velocity or load is altered between sets or exercises within the same session to increase muscle force expression [1]. This alternation might be done in several ways like decreasing or increasing loads between subsequent exercises to succeed in instant post-activation performance enhancement (PAPE), which refers to the temporary increase in sports performance following a high-intensity exercise, with the magnitude of this effect being influenced by various parameters [2–4]. The selection and order of the conditioning activity (CA), as well as an individual’s training background and experience, significantly influence the magnitude of the PAPE [5–7].

Studies have demonstrated that both resistance training with high load and low velocity, as well as exercises with low load and high velocity, such as plyometric or ballistic exercises, improve power performance [8]. However, adaptations resulting from these training methodologies seem to occur in different ways. High-load resistance exercises primarily contribute to an increase in maximum strength and power output, enhancing the force production component on the force-velocity curve [9]. Conversely, high-velocity exercises facilitate improvement in movement velocity [9]. Therefore, employing both approaches in athlete conditioning, such as CT, could allow for a broader range of adaptations. A growing body of evidence on CT has evaluated its effectiveness in improving athletic performance in team sports. For instance, a meta-analysis by Freitas et al. [10] reported improvements in medium-magnitude effect sizes in sprint performance and small effect sizes for vertical jump performance in team sports after CT intervention. Moreover, a meta-analysis by Thapa et al. [11] indicated that, compared to soccer training alone, CT induced moderate to large effect sizes improvements in linear sprinting time, vertical jump height, and sprinting with change of direction time in soccer players. However, the studies included in both meta-analyses did not investigate the effects of CT on the magnitude of the PAPE response.

Athletes with well-developed muscle strength and extensive experience in resistance training tend to exhibit a more pronounced PAPE effect while individuals with less training experience might still achieve the PAPE effect, but to a lesser extent [2, 6]. Chiu et al. [5] reported a significant enhancement in loaded squat jump among competitive-level athletes (n = 7; one soccer player, one triathlete, and 5 weightlifters) but not among their recreationally active counterparts. Similarly, Seitz et al. [6] found that stronger junior elite rug-by league players (back squat one-repetition maximum [1RM] > 2 × body mass) exhibited a higher PAPE response than the weaker ones (back squat 1RM < 2 × body mass). Moreover, the authors showed that stronger athletes demonstrated a significant PAPE effect earlier, at the 3^rd^ minute post-CA, whereas weaker athletes did so at the 6^th^ minute. Another study by Seitz et al. [7] found a moderately positive correlation between the PAPE response and maximal voluntary knee extension torque. This is supported by a recent study conducted by Guerra Jr. et al. [12], which reveals a significant and strong positive correlation between physical fitness level (Standard Ten score based on skinfold measures, squat jump height, agility T-test, Yo-Yo Intermittent Recovery Test level 1) and the magnitude of PAPE response (increase in countermovement jump [CMJ] height after a bout of plyometric exercises [45 jumps in total] and sled towing [15 m with 15% body mass]). Therefore, an increase in lower-body maximal strength or power output should result in a concurrent increase in PAPE magnitude.

It has been found that four weeks of resistance training decreased the recruitment threshold and increased the discharge rate of motor units when matched for the same relative force [13], which might explain why trained individuals exhibit greater neural excitation in response to high-intensity resistance exercises than untrained [6]. Moreover, there is a moderately positive correlation between the proportion of type II myosin heavy chain isoforms and maximal knee extension torque, where stronger individuals with a higher proportion of type II fibers attain a greater PAPE [7]. Another commonly cited explanation is that trained individuals are more fatigue-resistant [2, 6, 14]. This is relevant for eliciting the PAPE effect due to the coexistence of potentiation and fatigue induced by CA. As fatigue dissipates and the PAPE effect remains, performance enhancement occurs. This is also the reason why trained individuals experience PAPE earlier. To the best of the authors’ knowledge, no studies have exhaustively examined changes in PAPE responses due to changes in strength level caused by season or training intervention. Therefore, taking into account the available literature, it is plausible that an improved strength condition would also lead to a greater PAPE response.

For both the PAPE response and the success of CT, exercise selection and order play a crucial role. The selection of back squats has been successfully implemented to increase jump, sprint, and maximum strength during PAPE sessions and during 8 weeks of CT [15, 16]. Furthermore, hip thrust-based activation has been successfully used to improve subsequent sprint performance [17, 18, 19]. Questionable remains recommended loads for CT, where some research reports significant improvement after progressive to heavy loads (70–90% 1RM) [19], some after progressive fast exercise with low to moderate loads (30–60% 1RM) increase [16]. Although the ways how to induce PAPE are clearly stated [1], the question remains whether intensity and exercise selection differentiate the effects of long-term CT interventions.

Although the PAPE effect has been extensively studied in the realm of intensity and exercise selection, changes in PAPE response due to weeks of CT training intervention remain unknown. Therefore, this study aimed to determine the effect of 6 weeks of medium- and high-intensity CT on PAPE effect magnitude, changes in 5 and 30 m linear sprint, 5-0-5 change of direction (COD) test time, 1RM back squat and hip thrust, jumping performance (CMJ, drop jump [DJ], and broad jump [BJ]) in trained soccer players. It was hypothesized that both training interventions would result in considerable improvements in athletic performance, followed by an increase in the magnitude of the PAPE response.

## MATERIALS AND METHODS

### Research Design

**FIG. 1:**
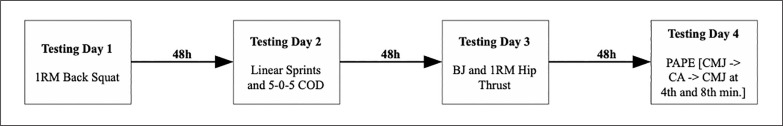
Pre- and post-intervention testing scheme. CMJ – countermovement jump; 1RM – one-repetition maximum; COD – change of direction; BJ – broad jump; PAPE – post-activation performance enhancement; CA – conditioning activity.

A randomized, single-blind, parallel-group intervention was conducted to compare the effects of medium- and high-intensity CT on maximum strength, jumping, and running performance, as well as the PAPE effect. Participants were randomly assigned to 6 weeks of medium- or high-intensity CT performed twice a week among soccer players during the off-season period. The medium-intensity training group (MED) performed CT with 65–70%1RM during the CA exercise, while the high-intensity training group (HIGH) used 80–85%1RM during the CA exercise (details below). Pre- and post-training assessments started 48–96 hours before the first and after the last training session and lasted for a week. The following condition tests were assessed: i) CMJ and 1RM back squat; ii) linear sprint and COD time; iii) BJ and 1RM hip thrust; iv) PAPE response assessed by CMJ performance after back squat CA (3 repetitions at 90%1RM) (Figure 1). All participants were familiar with the measurements performed because they constituted a part of the standard battery of tests during the off-season period.

### Participants

**FIG. 2:**
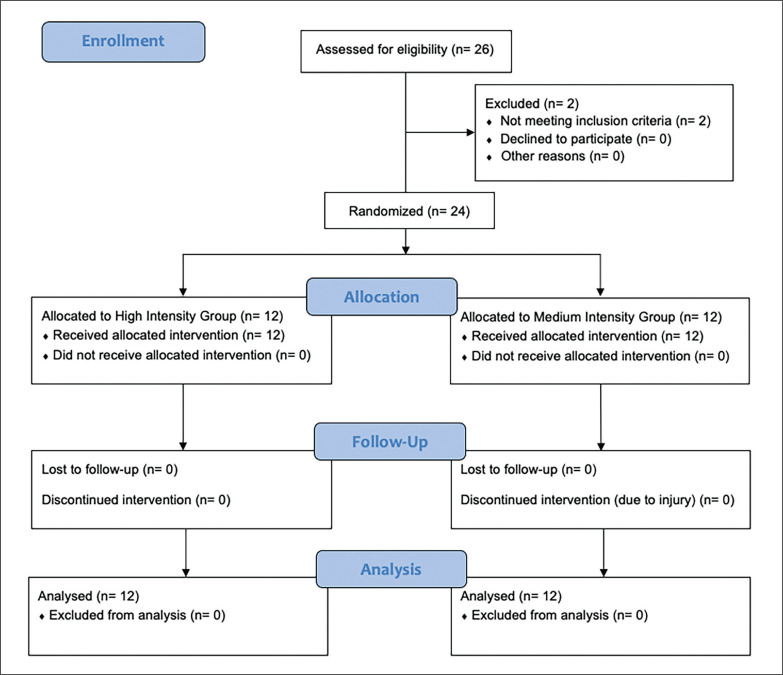
CONSORT flow diagram.

Twenty-four soccer players (national level as defined in McKay et al. [20]) selected from youth and second teams of clubs competing in the Ekstraklasa (the Polish first division) (age range: 18–19; body mass 72.8 ± 7.7 kg; height 175 ± 7 cm; soccer training experience: 5 ± 1 years; resistance training experience: 2 ± 1 years) participated in the experiment (Figure 2). The following criteria were used to select participants for the study: i) absence of neuromuscular and musculoskeletal disorders, ii) a minimum of two years of experience in resistance training, and iii) regular participation in soccer and resistance training for at least one year prior to the study. During the study, participants were instructed to maintain their typical dietary and sleep habits and to refrain from consuming stimulants and alcoholic beverages. In addition, they were instructed to refrain from performing additional resistance exercises during the study course and within 48 hours before the baseline examination to prevent fatigue. Participants were free to withdraw from the experiment at any moment, and they were provided with full details on the potential risks and benefits of the study before providing written informed consent. The study protocol was approved by Bioethics Committee for Scientific Research BLIND FOR PEER REVIEW and adhered to the ethical standards specified in the 2013 Helsinki Declaration.

A sample size estimation using G∗Power software (version 3.1.9.2, Dusseldorf, Germany) showed that to provide 80% power with a significance level of 0.05, correlation among repeated measures of 0.5 in this study design (n = 24; two-way repeated-measures ANOVA, within-between interaction) an effect size of approximately g = 0.3 will be required. This value of effect size was chosen according to findings from Thapa et al. [11] on the impact of CT on sprint, jump and COD ability of soccer players.

### Complex Training Program

The 6-week CT workout is described in Table 1. This training was characterized by performing sequences of three exercises in each training session, twice a week (72-h apart). Training sessions were compounded from six exercises performed in two CT tri-sets. The only difference between MED and HIG intensity was made during the first exercise of each complex, which had the role of CA exercise before the body mass exercise. The MED performed the first exercise with 65–70%1RM, while HIGH used 80–85%1RM with an intra-contrast rest interval of 120 s to following upper limb resistance exercise and 240 s intra-contrast rest to bodyweight vertical or horizontal power exercise (Table 1). Thus, each tri-set sequence consisted of CA as a resistance exercise (A1 or B1 in Table 1), then another exercise engaging upper-body muscles as an active rest interval (A2 or B2 in Table 1), followed by a bodyweight vertical or horizontal jumping exercise (A3 or B3 in Table 1). The implementation of an active rest interval was dictated to maintain a high training density [15]. Moreover, the comparison between medium and high intensities has been chosen, as both have been demonstrated to effectively induce the PAPE effect, along with enhancing maximum strength and power output [17, 21].

**TABLE 1:** Complex training program.

	Set [n]			Repetitions [n]	Intensity (%1RM)			Rest [s]

	Week 1–2	Week 3–4	Week 5–6	Week 1–6	Week 1–2	Week 3–4	Week 5–6	
A1 Back Squat	2	3	2	4 / 10^*^	80 / 65%^*^	80 / 65%^*^	85 / 70%^*^	120
A2 Bench Press	2	3	2	8	70%	70%	75%	120
A3 CMJ	2	3	2	3		BM		120
B1 Hip Thrusts	2	3	2	4 / 10^*^	80 / 65%^*^	80 / 65%^*^	85 / 70%^*^	120
B2 Chest-Supported Row	2	3	2	8	70%	70%	75%	120
B3 Broad Jump	2	3	2	3		BM		120

Rep – repetitions; 1RM – one repetition maximum; CMJ – countermovement jump; BM – body mass;

* medium intensity group.

### One-repetition Maximum Testing

The session began with a general warm-up consisting of 5 minutes of moderate-intensity stationary cycling, which was followed by dynamic stretching exercises targeting both single- and multi-joint movements, as well as 5 CMJ performed at 70% perceived effort. Subsequently, participants underwent back squat and bench press 1RM testing in randomized order. Both exercises were then performed with sets of 10, 6, 4, and 3 repetitions using 20 kg, 40%, 60%, and 80%, respectively, of the participant’s self-estimated 1RM. For each successive attempt, the load was gradually increased by 2.5 to 5 kg. Participants’ attempts were classified as unsuccessful if they were unable to attain parallel depth in the squat position or in the case of bench press, touch the chest and fully extend elbows. The greatest weight that could be lifted without assistance was recorded as the 1RM. All participants obtained their 1RM values within a maximum of five attempts, with five-minute recovery intervals between each attempt.

### Countermovement and Drop Jump Assessment

The performance of CMJ and DJ was assessed using force plates (Force Decks, Vald Performance, Australia), a validated and reliable device for measuring vertical jump kinematics [22]. For the CMJ, participants began with their arms on their hips in a standing position. Then, they were instructed to execute a downward movement to a depth of their choosing, followed by a powerful upward movement to reach the maximum jump height. Participants returned to the starting position after each jump and repeated the procedure twice for a total of two jumps. The following parameters, including jump height, relative peak power, and contraction time were evaluated. The greatest jump in terms of height was chosen for further analysis.

In the case of the DJ, participants started with their arms on their hips in a standing position and initiated the drop action. To do so, participants were instructed to “step off” the box (40 cm) one foot at a time and “jump up as quickly as possible after making contact with the ground, ensuring that the jump is as high as possible.” The participant was instructed to complete the contact and landing phases on the force plate. The participant’s jump was considered invalid if they elevated their feet during the jump flight, landed behind the force plate, or jumped off the box during the DJ. The procedure was completed with a total of two jumps, with the participant returning to the starting position after each jump. The jump height and contact time were evaluated, and the best attempt in terms of jump height was kept for further analysis.

The jumping height was determined from force impulse by using the following equation [23]:

Jump height=12⋅(TOV2/g)

where:

TOV – vertical velocity of the center of mass at take-off; g = 9.81 · sec^−2^

### 5-0-5 Change of Direction and Linear Sprint Time Assessment

For the 5-0-5 COD test, the participant sprinted as quickly as possible linearly from the starting point for 5 m, touching a ground line with their foot, and then returning to the starting point after a 180° COD. Times were recorded using timing photocells (SmartSpeed Pro, Fusion Sport, Coopers Plains, Australia) with checked validity and reliability for a 10 m sprint (intraclass correlation = 0.93) [24], with gates placed at 0 and 5 m. The height was set at approximately 1 m off the ground, corresponding to participants’ hip height to avoid the timing gates being triggered prematurely by a swinging arm or leg. The participants started with a front foot placed 0.3 m behind the first timing gate to prevent any early triggering of the photocells. Two attempts were performed with a turn on each leg in randomized order (four in total), and the best time was retained for further analysis. In the case of the linear sprint time assessment, the participant sprinted linearly from the starting point for 30 m with similar settings for the timing photocells; however, they were positioned at 0, 5, and 30 meters. The distances were selected to be the same as in previous CT research [16, 17, 19]. Two attempts were conducted, and the best time (5 m, 30 m) was retained for further analysis.

### Broad Jump Testing

The broad jump was performed as a bilateral standing long jump, and countermovement was permitted. A maximum of three trials was recorded where participants tried to jump as far as possible. This test has shown acceptable reliability, with an intraclass correlation of 0.97 [25]. Participants received instructions to bend their knees (choosing the depth of the bend themselves) and place their arms behind their torso. Following this, they generated a powerful thrust by extending their legs, propelling their arms forward, and executing a maximal jump for distance. The measurement of the distance jumped from the start line to the closest heel, in cm. Each participant undertook two attempts with a rest interval of 30 seconds between each jump. A better attempt was retained for subsequent analysis.

### Post-activation Performance Enhancement (PAPE) Testing

The PAPE testing session started with a standardized warm-up as described before in the 1RM testing section. Then the participants performed a set of 6 repetitions of the back squat at 50%1RM and another 3 repetitions at 70%1RM. Approximately 5 minutes later, two CMJ attempts were performed (as described in the countermovement jump assessment section). The CA began after an additional 5 minutes. The CA consisted of 3 repetitions of back squats at 90%1RM performed to approximately 90-degree knee flexion with a volitional movement tempo. Then, at the 4^th^ and 8^th^ minute after CA, the CMJ performance was re-tested. The best attempts in terms of jump height have been retained for further analysis.

### Statistical Analysis

All statistical analyses were performed using SPSS (version 25.0; SPSS, Inc., Chicago, IL) and were shown as mean with standard deviation (± SD), and median (Mdn) when a nonparametric test was used. Statistical significance was set at p < 0.05. The normality of data distribution was checked using Shapiro-Wilk tests.

The two-way ANOVAs (groups [MED; HIGH] × time points [pre-training; post-training]) were used to investigate the influence of training intensity on athletic performance, while an additional two-way ANOVAs (groups [MED; HIGH] × PAPE_% [pre-training; post-training]) were used to examine its impact of PAPE magnitude response. If a significant main effect or interaction was found, the post hoc tests with Bonferroni correction were used to analyze the pairwise comparisons. When a data distribution was violated Mann-Whitney U tests were used to compare differences between groups, while Wilcoxon signed-rank tests were used to compare pre- and post-training values within the group. The magnitude of mean differences was expressed with standardized effect sizes. Thresholds for qualitative descriptors of Hedge’s g was interpreted as < 0.20 “small,” 0.21–0.79 “medium,” and .0.80 as “large.”

Moreover, the Pearson correlation coefficient was used to analyze the relationship between pre- and post-intervention 1RM back squat, CMJ jump height, and the magnitude of PAPE response. Thresholds for qualitative descriptors of correlations were interpreted as follows: < 0.1 “trivial,” 0.1–0.3 “small,” 0.31–0.5 “moderate,” 0.51–0.7 “large,” 0.71–0.9 “very large,” and > 0.9 “nearly perfect.”

## RESULTS

A two-way ANOVA indicated a statistically significant interaction for the 5 m (F = 6.857; p = 0.016; *η*^2^ = 0.185) and 30 m sprint time (F = 5.009; p = 0.036; *η*^2^ = 0.185). The post-hoc analysis showed a significant decrease in post-training 5 m sprint time compared to pre-training in the MED (p = 0.004) and HIGH (p < 0.001) groups. Moreover, a significant decrease in post-training 30 m sprint time compared to pre-training (p = 0.001) was found in the HIGH group. Furthermore, a significantly shorter 5 m sprint time was found in the HIGH than the MED group (p = 0.001) at post-training. However, no statistically significant interactions were found for time in the COD test turned on the dominant leg (F = 1.571; p = 0.223; *η*^2^ = 0.067), and non-dominant leg (F = 1.75; p = 0.199; *η*^2^ = 0.074). No significant main effect of time or group was reported (Table 2).

**TABLE 2:** Linear sprint and 5-0-5 change of direction test time comparison between and within medium- and high-intensity training groups.

Test	Groups	Pre (95%CI)	Post (95%CI)	Pre vs. Post ES	Δ [%]
**5 m Linear Sprint Time [s]**	MED	1.13 ± 0.05 (1.09–1.16)	1.10 ± 0.06 (1.06–1.13)^*^	0.52	-2.7 ± 2.3
	HIGH	1.09 ± 0.04 (1.06–1.1)	1.02 ± 0.03 (1.0–1.04)^*†^	1.91	-6 ± 3.4

**30 m Linear Sprint Time [s]**	MED	4.33 ± 0.15 (4.26–4.4)	4.32 ± 0.14 (4.24–4.4)	0.07	-0.3 ± 1.2
	HIGH	4.28 ± 0.08 (4.21–4.35)	4.21 ± 0.12 (4.13–4.29)^*^	0.66	-1.6 ± 1.7

**COD-D Time [s]**	MED	2.25 ± 0.08 (2.2–2.3)	2.23 ± 0.11 (2.18–2.29)	0.2	-0.7 ± 2.8
	HIGH	2.30 ± 0.08 (2.25–2.35)	2.32 ± 0.07 (2.26–2.37)	0.26	0.74 ± 2.8

**COD-ND Time [s]**	MED	2.27 ± 0.09 (2.21–2.34)	2.26 ± 0.09 (2.2–2.32)	0.11	-0.7 ± 2.6
	HIGH	2.34 ± 0.13 (2.27–2.41)	2.27 ± 0.11 (2.21–2.33)	0.56	-3.1 ± 5.8

Mean ± SD; CI – confidence interval; ES – Hedges g effect size; MED – medium-intensity training group; HIGH – high-intensity training group; COD-D – change of direction test turned on the dominant leg; COD-ND – change of direction test turned on the non-dominant leg;

* significant difference compared to pre-training values within a group;

† significant difference between groups within corresponding time point.

A two-way ANOVA indicated no statistically significant interactions for 1RM back squat (F = 0.71; p = 0.409; *η*^2^ = 0.031), BJ length (F = 0.922; p = 0.347; *η*^2^ = 0.04), and CMJ height (F = 0.513; p = 0.481; *η*^2^ = 0.23). However, there were significant main effects of time to increase 1RM back squat (F = 6.387; p = 0.019; *η*^2^ = 0.225) and BJ length (F = 7.447; p = 0.012; *η*^2^ = 0.253) from pre- to post-training. There were no significant main effects of the group (Table 3).

**TABLE 3:** Maximum strength and jumping performance comparison between and within medium- and high-intensity training groups.

Test	Groups	Pre (95%CI)	Post (95%CI)	Pre vs. Post ES	Δ [%]
**1 RM Back Squat [kg]**	MED	110 ± 11 (103–117)	115 ± 16^*^ (105–125)	0.35	4.4 ± 7.9
	HIGH	105 ± 15 (95–115)	108 ± 16^*^ (97–118)	0.19	2.4 ± 5.3

**1 RM Hip Thrust [kg]**	MED	86 ± 8 (80–91)	89 ± 10 (82–95)	0.32	3.6 ± 5.3
	HIGH	80 ± 16 (70–90)	84 ± 14† (75–93)	0.26	5.4 ± 7

**Broad Jump [cm]**	MED	227 ± 18 (216–237)	239 ± 15^*^ (228–249)	0.7	2.9 ± 3.2
	HIGH	232 ± 15 (224–242)	242 ± 16^*^ (233–250)	0.62	1.3 ± 4

**CMJ Height [cm]**	MED	34.3 ± 4.2 (31.7–36.9)	34.8 ± 3.4 (32.4–37.2)	0.13	2.1 ± 8.2
	HIGH	37.3 ± 4.5 (34.7–39.9)	38.4 ± 4.5 (36–40.8)	0.24	3.2 ± 2.7

**CMJ Relative Peak Power Output [W/kg]**	MED	50.8 ± 4.5 (47.9–53.6)	51.8 ± 5.3 (48.4–55.1)	0.2	2.1 ± 5.7
	HIGH	57.5 ± 4.4 (54.7–60.3)	57.4 ± 6.2 (53.4–61.3)	0.02	-0.3 ± 7.2

**DJ Height [cm]**	MED	29.3 ± 3 (27.4–31.2)	29.4 ± 2.7 (27.7–31.2)	0.03	0.6 ± 4.6
	HIGH	31.7 ± 7.8 (26.7–36.7)	36.4 ± 8.2† (31.2–41.5)	0.57	16 ± 10.7‡

**DJ Contact Time [ms]**	MED	0.220 ± 0.031 (0.201–0.240)	0.223 ± 0.033 (0.202–0.244)	0.09	1.2 ± 2.3
	HIGH	0.244 ± 0.041 (0.217–0.270)	0.230 ± 0.023 (0.215–0.244)	0.41	-3.5 ± 17.7

Mean ± SD; CI – confidence interval; ES – Hedges g effect size; MED – medium-intensity training group; HIGH – high-intensity training group; ES – effect size; 1RM – one-repetition maximum; CMJ – countermovement jump; DJ – drop jump;

* significant difference compared to pre-training values within a group by ANOVA and Bonferroni post hoc test;

† significant difference compared to pre-training values within a group by Wilcoxon signed-rank test;

‡ significant differences in percentage change from pre- to post-training between groups by Mann-Whitney U test.

Wilcoxon signed rank test didn’t reveal significant differences in pre- to post-training values of 1RM hip thrust (p = 0.053; Mdn: 85 vs. 87.5 kg), CMJ peak power output (p = 0.272; Mdn: 50.75 vs. 50.60 W/kg), DJ height (p = 0.906; Mdn: 29.8 vs. 29.4 cm), DJ contact time (p = 0.414; Mdn: 0.217 vs. 0.219 ms) in the MED group. Similarly, there were no differences in CMJ peak power out-put (p = 0.844; Mdn: 56.95 vs. 59.30 W/kg) and DJ contact time (p = 0.23; Mdn: 0.240 vs. 0.229 ms) in the HIGH group. However, a significant increase from pre- to post-training in 1RM hip thrust (p = 0.035; Mdn: 78.75 vs. 87.50 kg) and DJ height (p = 0.002; Mdn: 31.25 vs. 34.90 cm) was revealed in the HIGH group. Mann-Whitney U test didn’t indicate significant differences in percentage change from pre- to post-training values between groups for 1RM hip thrust (p = 0.478; MED vs. HIGH: Mdn: 3.5 vs. 6.0%; ES = 0.28), CMJ peak power output (p = 0.443; Mdn: 3.5 vs. -1.0%; ES = 0.36), and DJ contact time (p = 0.219; MED vs. HIGH: Mdn: 0 vs. -5.5%; ES = 0.36) but the significantly lower percentage increase in DJ height (p < 0.001; MED vs. HIGH: Mdn: -0.5 vs. 14%; ES = 1.81) was found in MED than in HIGH.

### Post-Activation Performance Enhancement Response

Two-way ANOVAs indicated neither statistically significant interactions for PAPE response in CMJ height (F = 0.054; p = 0.818; *η*^2^ = 0.002) nor CMJ peak power output (F = 0.37; p = 0.549; *η*^2^ = 0.008), the main effect of time (pre- vs. post-training) for PAPE response in CMJ height (F = 0.586; p = 0.452; *η*^2^ = 0.01) and CMJ peak power output (F = 2.923; p = 0.101; *η*^2^ = 0.061), nor the main effect of the group for PAPE response in CMJ height (F = 0.723; p = 0.404; *η*^2^ = 0.019). However, a significant main effect of the group, indicating a higher PAPE response in the MED group compared to the HIGH group for CMJ peak power output, was observed at both pre- and post-CT intervention (F = 4.523; p = 0.045; *η*^2^ = 0.171) (Table 4).

**TABLE 4:** Comparison of post-back squat countermovement jump performance enhancement between and within medium- and high-intensity training groups.

Variable	Group	Pre training				Post training			

		Pre-CA (95%CI)	Post-CA (95%CI)	ES	Δ [%]	Pre-CA (95%CI)	Post-CA (95%CI)	ES	Δ [%]
**CMJ Height [cm]**	MED	34.3 ± 4.2 (31.7–36.9)	34.9 ± 3.7 (32.7–37.1)	0.15	2.1 ± 5.7	34.8 ± 3.4 (32.4–37.2)	35.8 ± 3.1 (33.3–38.2)	0.3	2.9 ± 4.32.9 ± 4.3

	HIGH	37.3 ± 4.5 (34.7–39.9)	37.4 ± 3.8 (35.2–39.7)	0.02	0.7 ± 4.1	38.4 ± 4.5 (36–40.8)	39.2 ± 5 (36.7–41.6)	0.16	1.9 ± 5.2

**CMJ Relative Peak Power Output [W/kg]**	MED	50.8 ± 4.5 (47.9–53.6)	51 ± 4.1 (48.4–53.5)	0.04	0.6 ± 5.1^*^	51.8 ± 5.3 (48.4–55.1)	52.9 ± 4.6 (49.5–56.2)	0.21	2.3 ± 3.7^*^

	HIGH	57.5 ± 4.4 (54.7–60.3)	55.4 ± 4.4 (52.8–57.9)	0.46	-3.7 ± 5.4	57.4 ± 6.2 (53.4–61.3)	57.2 ± 6.3 (53.8–60.5)	0.03	-0.3 ± 4.4

Mean ± SD; CA – conditioning activity; ES – Hedges g effect size; MED – medium-intensity training group; HIGH – high-intensity training group;

* significant difference compared to HIGH group.

No statistically significant correlations were found between athletic performance variables vs. the magnitude of PAPE response at pre- and post-intervention for the MED as well as HIGH group (Table 5).

**TABLE 5:** Correlations between athletic performance variables with the magnitude of the post-activation performance enhancement response

Variable	MED Group		HIGH Group	

	Pre-intervention PAPE	Post-intervention PAPE	Pre-intervention PAPE	Post-intervention PAPE
1RM-BS_PRE_	0.353	0.39	-0.277	0.224
1RM-BS_POST_	0.443	0.499	-0.206	0.308
1RM-HT_PRE_	-0.37	0.398	-0.553	-0.186
1RM-HT_POST_	-0.266	0.356	-0.506	-0.3
CMJ_PRE_	-0.48	-0.402	-0.277	0.043
CMJ_POST_	-0.029	-0.452	-0.116	-0.017
BJ_PRE_	0.026	0.067	-0.41	0.046
BJ_POST_	0.16	0.186	0.04	0.435
DJ_PRE_	-0.44	0.14	-0.367	-0.089
DJ_POST_	-0.321	0.379	-0.384	0.052
5 m_PRE_	0.443	0.138	0.56	0.309
5 m_POST_	0.251	0.153	0.114	-0.147
30 m_PRE_	0.208	0.165	0.323	-0.425
30 m_POST_	0.190	-0.016	-0.013	-0.617

MED – medium-intensity training group; HIGH – high-intensity training group; PAPE – post-activation performance enhancement; 1RM – one-repetition maximum; PRE – pre-intervention; POST – post-intervention; BS – back squat; HT – hip thrust; CMJ – countermovement jump; BJ – broad jump; DJ – drop jump.

## DISCUSSION

The aim of this study was to verify the hypothesis that CT will contribute to the improvement of athletic performance and enhanced PAPE responses in soccer players. The main finding of this study was that HIGH CT led to a significant improvement in 5 m, 30 m, 1RM back squat, 1RM hip thrust, BJ length, and DJ height, while MED led to improvements in 5 m, 1RM back squat, and BJ length. The HIGH group resulted in greater improvement in 5 m and DJ height compared to the MED group, and MED resulted in improved PAPE response while the HIGH group did not change it.

Findings in the improvement of strength and power following CT are in agreement with previous research [11, 26, 27]. For example, Gee et al. [26] demonstrated a significant increase in vertical jump height (CMJ with arm swing), as well as improvements in 10 m and 40 m sprint times, but not in the COD test (Arrowhead COD test) after 10 weeks of CT training in soccer players. Interestingly, despite the current study lasting for a shorter period of time (6 weeks), in the HIGH group, greater improvements were observed in jumping performance (0.24–0.57 vs. 0.21) and sprinting (0.66–1.91 vs. 0.21) in comparison to the findings of Gee et al. [26]. These might be attributed to the fact that in the study by Gee et al. [26], the training intervention occurred during the late pre-season to early competitive season, potentially influencing the post-testing, while our study was conducted in the off-season. As a result, participants in the study by Gee et al. [26] may have already experienced improvements due to their season training, making further enhancements more difficult to achieve. Moreover, in both studies, there was no significant improvement in COD; although, the performed tests differed significantly in terms of the number of direction changes and angles (one change with 180 degrees vs. three changes with degrees of around 90–135). However, Maio Alves and colleagues [27] did not find any performance increase also in the 505-test used in their study after 6 weeks of CT. This may indicate that COD performance isn’t solely dependent on maximum strength and power output but on other factors and may require specific training interventions.

To the best of the authors’ knowledge, this is the first study that compared moderate- vs high-intensity CT. In terms of training methods, typically higher performance increases are achieved with greater intensities [28, 29]. This effect was confirmed by this research with a specification that high intensity leads to greater improvement in DJ height and 5 m sprint performance compared to the medium intensity load. However, it is noteworthy that there were no statistically significant differences seen across the groups in terms of their 1RM back squat, hip thrust, and BJ length. The MED group showed comparable or slightly greater improvements in 1RM back squats (ES: 0.35 vs. 0.19), as well as BJ length (ES: 0.7 vs. 0.62). Recent studies have proven that interventions with moderate loads lead to similar gains in maximum strength [21, 30]. For instance, Montalvo-Pérez et al. [30] evaluated the effects of a 6-week training intervention at moderate vs. high-intensity resistance training and reported similar gains in a squat and split squat maximum strength and power. Therefore, although more research is required in the future, the available evidence indicates that moderate intensity may be an alternative approach to achieving similar strength and power gains with lower levels of generated neuromuscular fatigue and perceived exertion [21].

Contrary to the stated hypothesis, despite the significant increase in condition observed in various tests in this study, a significant increase in the PAPE response was noted only in the MED group. Nonetheless, it should be noted that during baseline measurements, the PAPE effect assessed by the increase in CMJ height in both groups was small (~2.1%, ES: 0.15 for the MED group, and ~0.7%, ES: 0.02 for the HIGH group). Meanwhile, the HIGH group reported a decrease in CMJ relative peak power output at baseline (~ -3.7%, ES: 0.46), while the MED group noted a trivial increase (~0.6%) and showed a significant upward trend in the magnitude of the PAPE response. After 6 weeks of intervention, both the MED and HIGH training groups showed PAPE effects for CMJ height, with improvements of ~2.9% for the MED group (ES: 0.3) and ~1.9% for the HIGH group (ES = 0.16). Although the high-intensity training led to significant improvements in maximal strength, jumping, and sprinting performance, the magnitude of the PAPE effect increase did not reach statistical significance. This is not consistent with the relationship between strength or physical fitness level and the magnitude of the PAPE response indicated in previous studies [5, 6, 12]. One possible explanation for this outcome could be the presence of training-induced fatigue. Despite implementing training volume tapering in the last two weeks, the approach remained consistent across both groups. Therefore, there might be a slightly higher fatigue level in the HIGH group compared to the MED group. On the other hand, the greater increase in the magnitude of the PAPE effect in the MED group may be attributed to the fact that the application of moderate-intensity allowed a higher movement velocity, which was closer to the velocity noticed during the CMJ. As noted by Behm and Sale [31], strength gains from a specific exercise mode may not necessarily be effectively transferred to another mode, and the improvements observed may decrease as the test velocity deviates from the training velocity. Therefore, further studies with variations in training length, frequency, volume, and movement velocity compared to those applied in this study, along with monitoring fatigue, are necessary to fully understand the effects of complex training on PAPE magnitude.

While the current study did not evaluate physiological parameters, it is worth noting that previous research indicates that the differences seen may be largely attributed to enhanced fatigue resistance and improved muscle contractile properties [32, 33]. Less fatigability following resistance training seems to be a result of muscle angiogenesis [32]. Enhanced muscle capillarization can lead to greater cellular metabolic control, energy and substrate supply, and finally more efficient energy consumption during muscle contractions [34]. Furthermore, a protective effect due to the frequently overloaded muscles called the repeated bout effect might also attenuate fatigue induced by a CA [35]. Additionally, a recent study examining the time course of adaptations following 6 weeks of resistance training appears to confirm potential neural adaptations [36]. The authors demonstrated an increase in maximal strength after 4 weeks and neural adaptation via increased motor-evoked potential [36]. Similarly, del Vecchio et al. [13] showed increases in motor unit discharge rate and decreases in the recruitment-threshold force of motor units. These mentioned neuro-muscular changes may also explain the overall increase in physical fitness of participants, both in terms of maximal strength and power abilities [37]. To validate these hypotheses, research should focus on neuromuscular characteristics such as contractile properties and neural excitation patterns, which would provide a deeper understanding of the physiological factors related to the expression of PAPE responses and application of CT strategies.

The observed physical fitness improvements in this study is consistent with the results of previous research evaluating CT in soccer players. For instance, Maio Alves et al. [27] revealed similar enhancements in the 5 m sprint performance (by -7%) and no statistically significant changes in CMJ height (by +2.4%). Additionally, there were no differences in the 5-0-5 test time after 6 weeks of CT performed twice a week by elite soccer players. Similarly, the meta-analysis by Freitas et al. [10] indicated greater improvement in sprint performance (ES = 0.73) compared to vertical jump height (ES = 0.34) following CT in team sports.

Regarding maximal strength, the improvement observed in this study for back squat and hip thrust 1RM was lower than that reported in a study by Rodriguez Rosell et al. [38] with a similar protocol. The authors of that study demonstrated a significant increase in 1RM back squat (+13.4%, from 91.8 ± 14.7 to 104.4 ± 17.8 kg; ES = 0.72) after 6 weeks of CT performed twice a week by adult soccer players. However, it should be noted that the participants in the current study had a higher baseline back squat 1RM compared to the participants in the Rodriguez Rosell et al. study [38] (110 and 105 kg for MED and HIGH groups, respectively). The possibility of physical fitness development often plateaus or diminishes when athletes reach higher levels of performance [39]. Consequently, the observed enhancements in the aforementioned tests among the participants in this research were comparatively less pronounced than those reported in Rodriguez Rosell et al. study [38]. In summary, a 6-week CT performed twice a week is suitable for inducing significant improvements in lower-body maximal strength, linear sprinting, and jumping ability in soccer players.

In order to properly interpret the findings of this research, it is necessary to take into account the inherent drawbacks associated with it. It should be noted that the training intervention lasted for 6 weeks and was conducted twice a week, thus further research with different training volumes would be beneficial. Additionally, it’s important to acknowledge that only soccer players participated in the study, so extrapolating these results to other groups should be done cautiously. Finally, we did not assess any underlying mechanisms behind the observed improvements in physical fitness; therefore, we cannot speculate on the contribution of post-activation potential or another physiological response [40].

In summary, soccer coaches and practitioners could employ 6 weeks of either medium or high-intensity CT to enhance jumping performance, linear speed and lower-body maximum strength among soccer players. The improvement of COD remains questionable, as it does not appear to be directly enhanced with CT. CT in soccer might be rather used in moderate-intensity training to expect better PAPE response.

## CONCLUSIONS

CT improves the soccer player’s strength and power but does not improve COD performance. High-intensity CT is more effective in increasing acceleration and drop jump performance compared to medium-intensity training. Medium intensity results in an improved PAPE response, whereas the HIGH group does not exhibit a change. No significant relationships were observed between the analyzed athletic performance variables and the magnitude of PAPE response.

## Data Availability

The data that support the findings of this study are available on request from the authors.
